# A peptide factor secreted by *Staphylococcus pseudintermedius* exhibits properties of both bacteriocins and virulence factors

**DOI:** 10.1038/srep14569

**Published:** 2015-09-28

**Authors:** Benedykt Wladyka, Marcin Piejko, Monika Bzowska, Piotr Pieta, Monika Krzysik, Łukasz Mazurek, Ibeth Guevara-Lora, Michał Bukowski, Artur J. Sabat, Alexander W. Friedrich, Emilia Bonar, Jacek Międzobrodzki, Adam Dubin, Paweł Mak

**Affiliations:** 1 Department of Analytical Biochemistry, Faculty of Biochemistry, Biophysics and Biotechnology, Jagiellonian University, 30-387 Krakow, Poland; 2 Department of Cell Biochemistry, Faculty of Biochemistry, Biophysics and Biotechnology, Jagiellonian University, 30-387 Krakow, Poland; 3 Department of Microbiology, Faculty of Biochemistry, Biophysics and Biotechnology, Jagiellonian University, 30-387 Krakow, Poland; 4 Malopolska Centre of Biotechnology, Jagiellonian University, 30-387 Krakow, Poland; 5 Institute of Physical Chemistry, Polish Academy of Sciences, 01-224 Warsaw, Poland; 6 3rd Department of General Surgery, Jagiellonian University Medical College, 31-008 Krakow, Poland; 7 Department of Medical Microbiology, University of Groningen, University Medical Center Groningen, 9713 GZ Groningen, The Netherlands

## Abstract

*Staphylococcus pseudintermedius* is a common commensal bacterium colonizing the skin and mucosal surfaces of household animals. However, it has recently emerged as a dangerous opportunistic pathogen, comparable to *S. aureus* for humans. The epidemiological situation is further complicated by the increasing number of methicillin-resistant *S. pseudintermedius* infections and evidence of gene transmission driving antibiotic resistance between staphylococci colonizing human and zoonotic hosts. In the present study, we describe a unique peptide, BacSp222, that possesses features characteristic of both bacteriocins and virulence factors. BacSp222 is secreted in high quantities by *S. pseudintermedius* strain 222 isolated from dog skin lesions. This linear, fifty-amino-acid highly cationic peptide is plasmid-encoded and does not exhibit significant sequence similarities to any other known peptides or proteins. BacSp222 kills gram-positive bacteria (at doses ranging from 0.1 to several micromol/l) but also demonstrates significant cytotoxic activities towards eukaryotic cells at slightly higher concentrations. Moreover, at nanomolar concentrations, the peptide also possesses modulatory properties, efficiently enhancing interferon gamma-induced nitric oxide release in murine macrophage-like cell lines. BacSp222 appears to be one of the first examples of multifunctional peptides that breaks the convention of splitting bacteriocins and virulence factors into two unrelated groups.

*Staphylococcus pseudintermedius* is reported as a commensal bacterial species colonizing the skin and mucosal surfaces of household animals, especially dogs. However, as is typical for an opportunistic pathogen, the species is responsible for diseases such as pyoderma, otitis externa, and postoperative infections^1^,^2^. Most importantly, methicillin-resistant *S. pseudintermedius* (MRSP) has recently emerged as a significant problem in veterinary medicine and entails further consequences for humans, as the gene driving the drug resistance is highly mobile and can be transferred between different staphylococcal species, including *S. aureus*^1^,^3^. Moreover, the transmission of *S. pseudintermedius* and *S. aureus* between human and zoonotic hosts is evident and has resulted in severe infections in humans caused by MRSP^4^,^5^. The situation is even more complicated because the proper identification of *S. pseudintermedius* requires genetic techniques that are seldom applied in routine diagnostics^4^,^6^. Of note, prior to the discrimination of this bacterium as a novel species, *S. pseudintermedius* has been referred to as *S. intermedius* or even *S. aureus*^7^,^8^. Consequently, the importance of this bacterium as a pathogen appears to be underestimated^9^. Such underestimation has been reflected in recent genomic data clearly indicating that *S. pseudintermedius* is equipped with genes homologous to those encoding virulence factors and regulatory systems characteristic of *S. aureus*^10^,^11^. Indeed, the pathogenic potential of *S. pseudintermedius* in household animals is emerging and comparable to the *S. aureus* threat in humans^12^.

A necessary prerequisite for any bacterial infection is successful host colonization followed by adequate management of the host immune system. This ability is of special importance for opportunistic pathogens such as staphylococci. For example, *S. aureus* possesses an array of factors facilitating adhesion to host cells. The binding is mediated by surface-exposed proteins such as fibronectin-binding proteins, collagen-binding protein, fibrinogen-binding proteins, and clumping factors^13^. Moreover, proteins released into the extracellular milieu such as coagulase and von Willebrand factor-binding protein also facilitate colonization^14^. Upon colonization, the bacteria can spread from the initial niche by modulating the immune response of the host. This process is triggered by the secretion of enzymes that induce remodelling of the *S. aureus* cell surface and cause deregulation of the host’s defences. The latter event is also accomplished by the lysis of the host blood cells by nonenzymatic toxins, including haemolysins, leucocidins, and phenol-soluble modulins (PSMs), as well as by immunomodulatory chemoattractants such as chemotaxis inhibitory protein of staphylococci (CHIPS) and formyl peptide receptor like-1 inhibitory protein (FLIPr)^15^,^16^,^17^. In addition, especially during colonization, the pathogen must also address the challenge of other bacteria residing in the same ecological niche. This task is commonly attributed to bacteriocins, which are ribosomally synthesized peptides or proteins that are able to kill closely related bacteria^18^. Both types of staphylococci, namely those considered to be exclusively commensal and those that are opportunistic pathogens, were demonstrated to produce bacteriocins, which indicates their importance in maintaining the microbiome of the skin and mucosal surfaces^19^,^20^,^21^. Interestingly, despite their undeniable roles in successful host colonization, virulence factors and bacteriocins have been considered separately, with much less attention given to the latter. Presumably, this division is due to the lack of evidence indicating the cross-activity of virulence factors with other bacteria, as well as the nontoxic nature of staphylococcal bacteriocins against eukaryotic hosts.

In this article, we describe the isolation and characterization of a unique peptide, BacSp222, which possesses features characteristic of both bacteriocins and virulence factors. The peptide is produced and secreted in high quantities by *S. pseudintermedius* strain 222, which was isolated from dog skin lesions. BacSp222 is bactericidal for related staphylococci at micromolar concentrations. However, at slightly higher doses, it also demonstrates cytotoxic activity towards eukaryotic cells. Moreover, at very low and nonbactericidal concentrations, the peptide demonstrates modulatory properties by efficiently enhancing interferon gamma-induced nitric oxide release in murine macrophage-like cell lines.

## Results

### *Staphylococcus pseudintermedius* strain 222 produces a novel peptide bacteriocin

**Figure 1:**
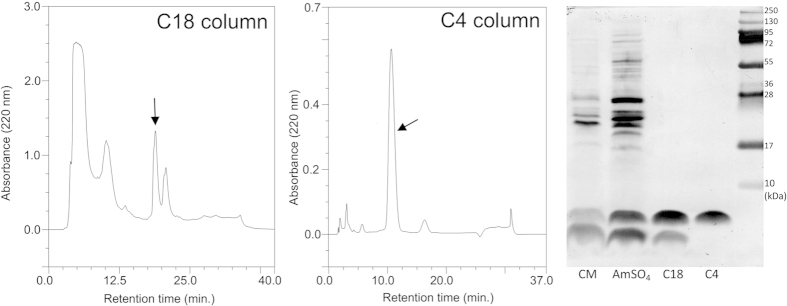
Purification of BacSp222. Culture media (CM) have been precipitated with ammonium sulphate (AmSO_4_) and further purified by two sequential steps of RP-HPLC chromatography on C18 and C4 columns (bacteriocin peak is marked by an arrow). The composition of the fractions representing each step of purification is illustrated by SDS-PAGE.

**Figure 2:**
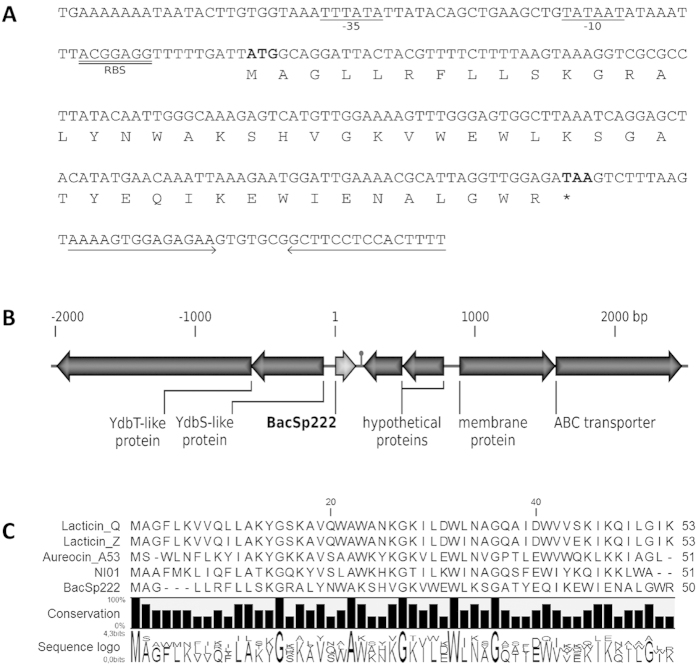
Analysis of the BacSp222 sequence. (**A**) Sequence of the *bacSp222* structural gene. The start and stop codons of the coding sequence are bolded. Upstream, a putative promoter −35 and −10 regions and a putative ribosome binding site (RBS) are underlined. Downstream, the reverse horizontal arrows indicate a putative transcription terminator. (**B**) Genetic organization of the *bacSp222* gene cluster. Nucleotide numeration with respect to the first nucleotide in *bacSp222* (denoted as 1 on the scale above the scheme). An inverted repeat downstream *bacSp222* is marked with a hairpin. ORFs are marked with arrows in the scale. (**C**) Alignment of the amino acid sequence of BacSp222 and similar bacteriocins: lacticins Q and Z, aureocin A53, as well as epidermicin NI01 (prepared using CLC Workbench software).

Recently, we have established a method based on the restriction fragment length polymorphism (RFLP) technique, which allows the determination of the *Staphylococcus* species within the *Staphylococcus intermedius* group (SIG)^6^. During the method validation, our attention was focused on *S. pseudintermedius* strain 222, which was isolated from skin lesions in a dog; this bacteria was found to produce an agent inhibiting the growth of methicillin-resistant *S. aureus* (MRSA) strain USA300. The bacteriocin, termed BacSp222, was purified from the culture fluid using a three-step procedure comprising precipitation with ammonium sulphate and two steps of reversed-phase chromatography. The peptide obtained was homogenous, as evidenced by a single band at approximately 5 kDa on SDS-PAGE gel (Fig. 1). The purification procedure yielded 0.7 mg of bacteriocin from 1 l of culture supernatant (5% overall yield; Supplementary Materials Table S2). MALDI-ToF analysis indicated that the molecular mass of the peptide was 5921.92 Da. BacSp222 contains a modified N-terminal because it was impossible to directly sequence the peptide using Edman degradation. After the deformylation procedure, the 50-mer amino acid sequence was determined: MAGLLRFLLSKGRALYNWAKSHVGKVWEWLKSGATYEQIKEWIENALGWR (deposited under the accession number C0HJT1, UniProt Knowledgebase). Assuming the presence of a N-formyl group at the N-terminus, the theoretical molecular mass of such a peptide is 5921.89 Da, which perfectly agrees with the mass assessed by MS. BacSp222 is a highly cationic peptide (theoretical pI = 10.09) that is rich in tryptophan residues. Using BLAST search, we were unable to find statistically significant similar peptide sequences. However, we found certain arbitrary similarities to bactericidal peptides such as lacticins Q and Z from *Lactococcus lactis*^22^,^23^,^24^,^25^, epidermicin NI01 from *S. epidermidis*^21^, and aureocin A53 from *S. aureus* 19,26,27 (Fig. 2C). Prediction of the BacSp222 secondary structure using CLC Main Workbench software suggested the presence of two alpha helices (2–31 and 36–47 amino acid residue), which is in agreement with the experimentally determined CD spectrum and secondary structure motifs calculations (48.4% and 52.8% alpha-helix, 9.3% and 4.6% beta structure, 38.5% and 6.6% turns, and 3.8% and 35.9% random structure using Prot and Yang reference spectra sets, respectively) (Supplementary Materials Fig. S1A). The hydrodynamic diameter of the BacSp222 molecule estimated by DLS was 3.68 nm (Supplementary Materials Fig. S2). The value appears to be approximately 1 nm too high, based on the diameters of other peptides with a similar molecular weight^28^, suggesting an elongated shape of the BacSp222 molecule or, possibly, the formation of dimers. Unfortunately, the estimation of BacSp222′s structure in solution by gel filtration was impossible, most likely due to strong interactions between the bacteriocin and the column packing, which resulted in nonspecific shifting of the retention time.

### BacSp222 is plasmid-encoded

*S. pseudintermedius* strain 222 carries a plasmid, termed here p222, consisting of 14 857 base pairs, as determined using next-generation sequencing. Within the plasmid sequence, we identified an open reading frame (ORF) encoding BacSp222. The ORF contains no secretion signal for the peptide. Upstream the ORF, a putative promoter sequence followed by a ribosome binding site was found, whereas an inverted repeat sequence indicating a putative terminator was identified downstream (Fig. 2A). The structural gene for BacSp222 is flanked by two ORFs with significant homology to YdbS and YdbT proteins from *Bacillus subtilis* involved in immunity against bacteriocins^29^ and two potentially clustered partially overlapping ORFs encoding hypothetical proteins. Moreover, upstream of the latter, two additional ORFs encoding a membrane protein and a protein with significant similarity to the epidermicin ABC transporter were identified^30^. Hence, besides *bacSp222*, p222 carries the genes putatively responsible for secretion of the bacteriocin, as well as self-protection (immunity) (Fig. 2B).

### BacSp222 is bactericidal and lytic towards gram-positive bacteria

**Figure 3:**
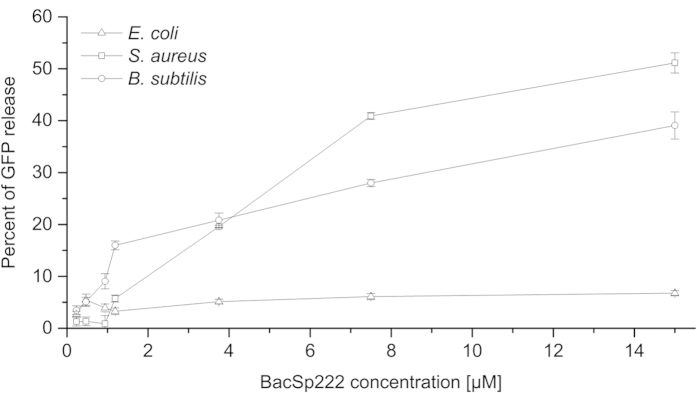
Release of GFP from *B. subtilis*, *S. aureus*, and *E. coli* cells transformed with GFP-expressing plasmids after 1 h of incubation with BacSp222. The percent GFP release is defined as the fraction of fluorescence in the clarified supernatant relative to the fluorescence of the non-lysed bacteria suspension. The points represent the average values from three independent experiments ± SD.

The bactericidal activity of BacSp222 was determined for a range of gram-positive and gram-negative bacteria, as well as for the fungal pathogen *C. albicans*, using the microdilution assay (Table 1). Of the evaluated species, neither the gram-negative species nor the fungi were susceptible to the bacteriocin, even at the highest concentration tested (100 μM). In contrast, BacSp222 effectively inhibited the growth of 15 strains of gram-positive bacteria at MIC values ranging from 0.11 μM (for *Micrococcus luteus*) to 7.8 μM (for *Streptococcus pyogenes*). For a majority of the tested *Staphylococcus* strains, the MIC values were near 1 μM. Interestingly, the bacteriocin-producing strain, *S. pseudintermedius* 222, was also susceptible to BacSp222 (MIC 2.1 μM); however, the growth of a nonproducer strain *S. pseudintermedius* LMG 22219 was inhibited by a bacteriocin concentration more than ten-fold lower (MIC 0.16 μM). Remarkably, a synthetic BacSp222 without N-terminal formylation exhibited moderately increased (123 ± 4.5% of the activity of the native BacSp222) activity against the indicator strain *B. subtilis* ATCC 6633, whereas the removal of the formylated N-terminal methionine with cyanogen bromide resulted only in a negligible decrease of the bactericidal potential (93 ± 6.5% of the activity of the native BacSp222). BacSp222 is bactericidal because the incubation of *B. subtilis* and *S. aureus* (1 × 10^4^ CFU) with 1 μM bacteriocin for 30 minutes resulted in the complete absence of the colonies after plating on TSA (data not shown). BacSp222 is lytic for susceptible cells because the dose-dependent leakage of GFP was observed from recombinant *B. subtilis* and *S. aureus* but not for bacteriocin-resistant *E. coli* (Fig. 3).

### BacSp222 is a thermostable peptide and highly resistant to proteases

Assuming a linear, alpha-helical structure for BacSp222, the peptide’s susceptibility to proteases was evaluated. To this end, the peptide was treated with proteinase K, staphylococcal extracellular proteases (V8 and StpC), and digestive enzymes (pepsin and trypsin), as well as proteases released during degranulation of neutrophils (elastase and cathepsin G). Strikingly, the activity of BacSp222 was not significantly reduced even after 3 hours of incubation with the enzymes (Supplementary Materials Fig. S3). The bacteriocin was also exceptionally stable during incubation at elevated temperatures, as further evidenced using CD spectroscopy by the lack of changes in ellipticity at 220 nm, up to 90 °C (Supplementary Materials Fig. S1B). Only prolonged boiling and autoclaving decreased significantly the peptide’s activity against the indicator strain (64 ± 2.0% and 55 ± 8.8%, respectively) (Supplementary Materials Fig. S3).

### BacSp222 production starts at logarithmic phase of growth

To assess the kinetics of BacSp222 production, we monitored the amount of the bacteriocin in the culture medium during *S. pseudintermedius* 222 growth using HPLC. BacSp222 accumulated in the medium starting from the 4^th^ hour postinoculation, and its levels increased until the late logarithmic phase of growth, reaching a concentration of nearly 13 μM (79 mg/l) (Supplementary Materials Fig. S4). This result is in agreement with the transcription of *bacSp222* assessed by qRT-PCR. The transcript was detected from the 2^nd^ to 12^th^ hour postinoculation (data not shown). The final level of bacteriocin in the late culture is much higher than its MIC dose towards producer cells (2.1 μM), but this inconsistency is a consequence of the fact that the MIC dose is estimated at much lower concentrations of cells than those observed after 24 hours of cultivation (10^5 ^CFU/ml for MIC vs. 10^9 ^CFU/ml for late culture). Moreover, *S. pseudintermedius* 222 streaked on a plate with a soft nutrient agar containing the same bacteria after overnight incubation at 37 °C resulted in the lack of an inhibition zone, clearly indicating the resistance of the producing bacteria to the toxic effects of BacSp222 (Supplementary Materials Fig. S5).

### BacSp222 is cytotoxic to eukaryotic cells

**Figure 4:**
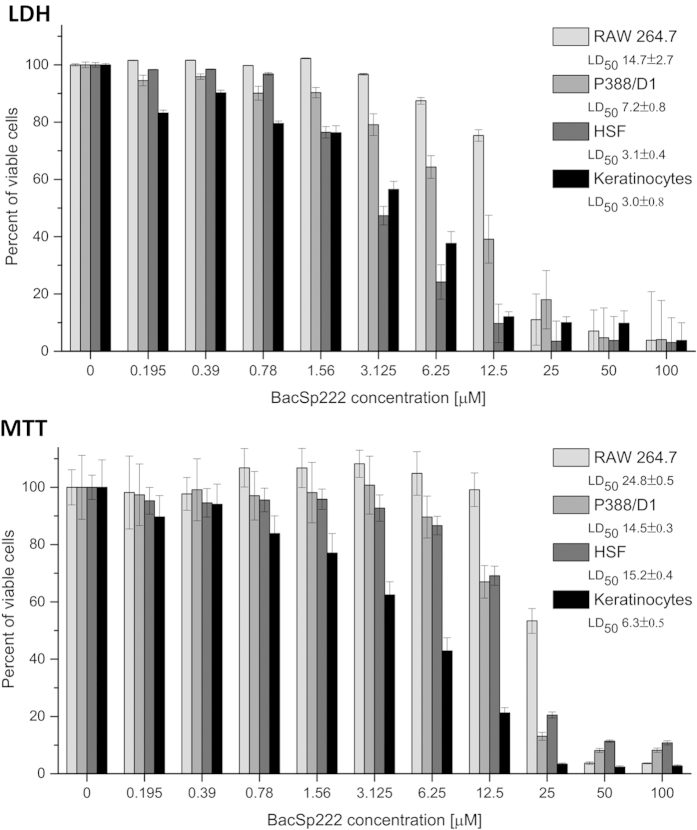
Cytotoxic activity of BacSp222 against different cells, as evaluated by LDH and MTT assays. LD50 values are provided in the inserts. The bars represent the average values from three independent experiments ± SD.

**Figure 5:**
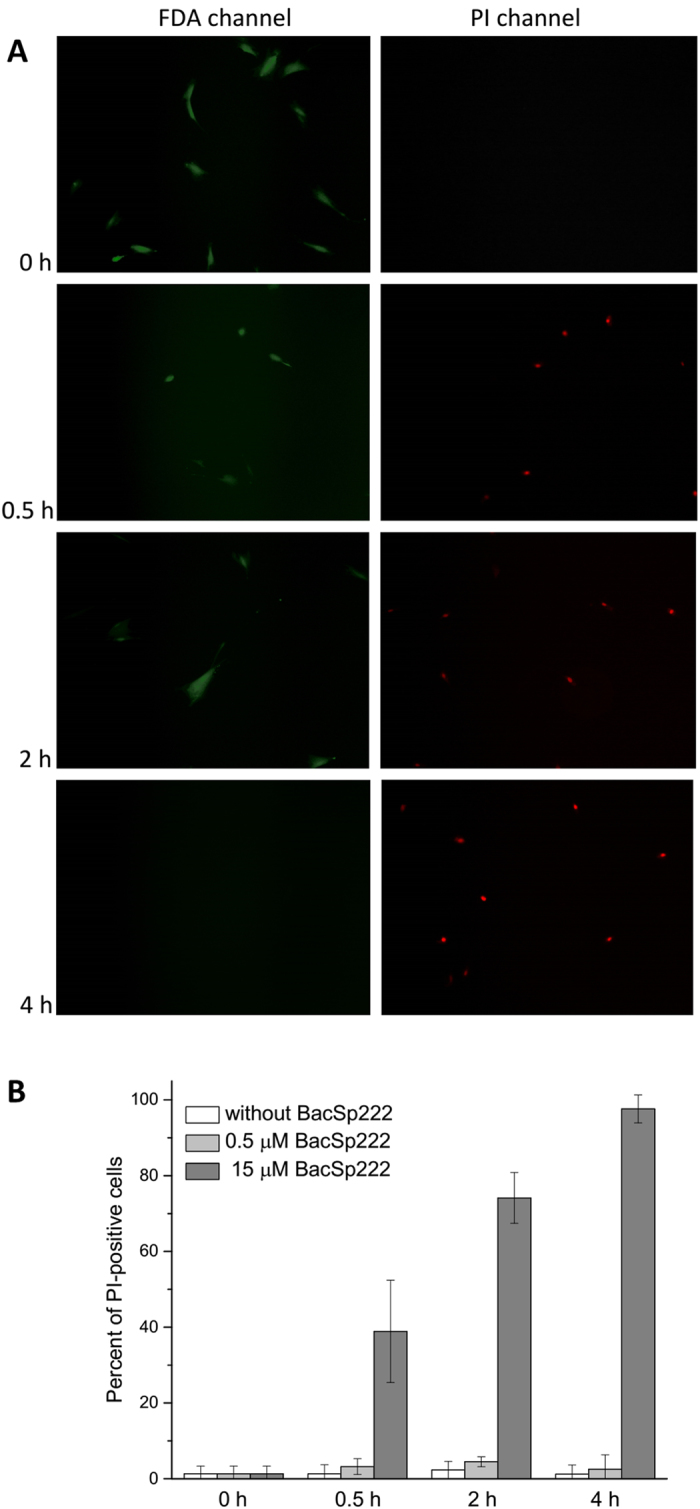
Disruption of cell membrane integrity in HSF cells by BacSp222. The cells were incubated for 0, 0.5, 2, or 4 h in cell media containing 0.5 or 15 μM BacSp222, as well as fluorescein diacetate (FDA, live cells) and propidium iodide (PI, dead cells). The cells were visualized using a fluorescence microscope (**A**), and the percentage of dead cells was estimated (**B**). The bars represent the average values from three independent experiments ± SD.

Because BacSp222 is a potential bactericidal agent, we then verified its safety against eukaryotic cells. To this end, we treated human erythrocytes with the bacteriocin up to 100 × MIC against *S. aureus* strains. At the highest concentration (100 μM), BacSp222 caused 40% lysis; however, at approximately 10 × MIC (12.5 μM, comparable to the level during *in vitro* production), only 1.82 ± 0.28% of the erythrocytes were lysed (Supplementary Materials Fig. S6). Because such concentrations of the bacteriocin in the bloodstream would be likely impossible, the potential systemic cytotoxicity is rather limited. However, one cannot exclude the possibility that BacSp222 may reach local concentrations comparable to those achieved *in vitro*. Moreover, except in the case of deep wound infections, the cells mostly likely to be first affected by the bacteriocin would be those of the epidermis and innate immune system rather than erythrocytes. Therefore, we incubated human skin keratinocytes, HSF, and two murine monocyte/macrophage cell lines with increasing concentrations of BacSp222. The remaining cell viability was determined both by the assessment of cell membrane integrity (LDH release assay) and using a metabolic test (ability to reduce MTT). Surprisingly, the addition of the peptide resulted in a sigmoidal, dose-dependent cytotoxic effect (Fig. 4). A similar effect was also observed for HeLa and adipose-derived stem cells (ASC, Supplementary Materials Fig. S7). In the LDH test, the calculated 50% lethal dose (LD_50_) was approximately 3 μM for keratinocytes and HSF, whereas it was slightly higher for murine macrophage-like cell lines. The same trend was observed using the MTT test, but the values were about two-fold higher. Independently of the LDH assay, the ability of BacSp222 to disrupt the cell membrane integrity was confirmed through the observation of HSF in cell media containing propidium iodide (PI) (Fig. 5). After 0.5, 2 or 4 hours of incubation with the bacteriocin, 39 ± 13.5, 74 ± 6.7, and 97 ± 3.7% of the cells were PI-positive, respectively, whereas no such cells were observed in the control set-up.

### BacSp222 at low concentrations induces production of nitric oxide

**Figure 6:**
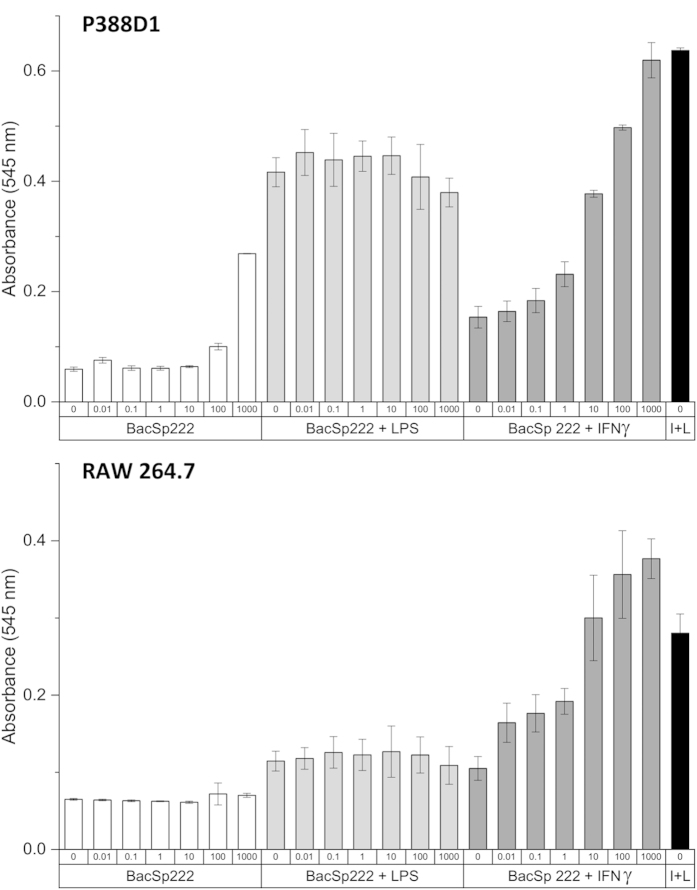
The analysis of the effect of BacSp222 on NO synthesis by P388D1 and RAW264.7 cells. The cells were cultured for 24 h in DMEM supplemented with 2% FBS without BacSp222 (controls) or i) with BacSp222 at different concentrations (0.01–1000 nM) alone or in combination with ii) LPS (100 ng/ml) or with iii) IFNγ (10 ng/ml). The positive control for NO production was cells stimulated with LPS and IFNγ (denoted as I+L). After incubation, the nitrite levels in the culture media were determined spectrophotometrically at 545 nm. The bars represent the average values from three independent experiments ± SD.

BacSp222 is an N-formylated, highly cationic, and tryptophan-rich peptide. Such features, to some extent, make the molecule similar to bacterial agonists of cellular formyl-peptide receptors (FPRs). Such receptors are expressed on phagocytic cells and are involved in sensing chemoattractants released by bacteria, as well as host-derived N-formylated peptides^31^,^32^. Nevertheless, at low to sub-cytotoxic concentrations (10 pM–5 μM), BacSp222 was not able to stimulate such receptors, as evaluated by recombinant CHO-K1 cells overexpressing FPRL-1 (data not shown). However, at comparable concentrations, the peptide demonstrated the ability to effectively stimulate production of nitric oxide (NO) through the activation of induced nitric oxide synthase (iNOS). To analyse the effect of BacSp222 on NO production by iNOS, the murine cell lines P388D1 and RAW264.7 were used. Because iNOS expression is controlled by very complex mechanisms and a single agent may induce NO synthesis only in some cell types, we incubated the cells with BacSp222 alone and in combination with LPS or IFN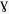 as costimulators. We observed that both cell lines slightly differed in their response to BacSp222 (Fig. 6). Only P388D1 cells were able to produce NO after exposure to BacSp222 alone (at 100 and 1000 nM). BacSp222 did not affect LPS-induced NO production. However, when the cells were treated with BacSp222 (at nanomolar concentrations) together with IFN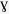, both P388D1 and RAW264.7 released NO in a dose-dependent manner. This pronounced effect suggests strong synergy between BacSp222 and IFN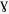 on the induction of iNOS expression in murine macrophage cell lines.

## Discussion

We have described a unique peptide, BacSp222, produced by *S. pseudintermedius*. The peptide is bactericidal towards gram-positive bacteria, including staphylococci; however, its activity towards mammalian cells extends its role from a novel bacteriocin to a multifunctional virulence factor-like that is able to modulate the activity of host immune system cells. *S. pseudintermedius* is considered to be the canine counterpart of *S. aureus* in humans. The bacteria’s pathogenicity has been neglected until recently, when proper identification techniques have become more available, allowing unambiguous differentiation of the bacterium from the other members of the *S. intermedius* group (SIG), as well as from *S. aureus*. Despite the emergence of *S. pseudintermedius* as a pathogen, the minimal amount of information concerning its virulence factors have been obtained mostly from analyses of genomic data. However, *S. pseudintermedius* has been reported to produce haemolysins, proteolytic enzymes, thermonuclease, coagulase, exfoliative toxins, leucotoxins, and enterotoxins. Moreover, the species possesses the ability to form a biofilm and to bind to fibrinogen, fibronectin, and cytokeratin. Furthermore, similarly to *S. aureus*, *S. pseudintermedius* possesses an accessory gene regulator (*agr*), which is an important quorum-sensing system that plays a key role in the regulation of virulence during infection^33^,^34^. Aside from a single study, nothing is known about bactericidal agents produced by *S. pseudintermedius*^35^.

BacSp222 does not demonstrate statistically significant sequence similarity to any known protein or peptide. However, based on its biological (bactericidal) activity, the size of the molecule, the formylation of N-terminal methionine, the abundance of tryptophan residues, and the lack of cysteines, BacSp222 is moderately similar to bacteriocins such as lacticins Q and Z from *Lactococcus lactis*^22^,^23^,^24^,^25^, epidermicin NI01 from *S. epidermidis*^21^,^30^, and aureocin A53 from *S. aureus*^19^,^26^,^27^ (Fig. 2C). All of these bacteriocins are linear 51–53 amino acid peptides, and, similar to BacSp222, highly cationic. However, the composition of charged amino acids in BacSp222 differs slightly. The peptide contains only five lysine residues, whereas the other bacteriocins contain 8 to 10. Moreover, BacSp222 has four glutamic acids, while the others possess only up to two. However, these acidic residues are compensated by three arginines. Lacticins, NI01 peptide, and aureocin A53 lack such residues. This composition may influence the overall shape of BacSp222, which was predicted to be elongated according to the DLS results and which contrasts with the structure of epidermicin and lacticins that are predicted preliminarily to be α-helical globular molecules^21^,^30^.

BacSp222 is plasmid encoded. The same localization is observed for the genes encoding aureocin A53 and epidermicin NI01^19^,^21^,^27^,^30^. In contrast, lacticins Q and Z are chromosomally encoded^22^,^23^. Regardless of the localization, the structural gene for the bacteriocins is similar. In all cases, the gene is monocistronic and does not code for a leader or *sec*-dependent signal peptide. However, the organization of genes putatively involved in BacSp222 secretion and self-immunity resemble neither those of staphylococcal nor lactococcal bacteriocins. In the former, the bacteriocin structural gene is preceded by two genes encoding YdbS and YdbT proteins involved in self-immunity, whereas in the case of *bacSp222*, the respective genes are also upstream but on the reverse strand. Downstream of the BacSp222 structural gene, in the opposite orientation, two open reading frames (ORFs) homologous to those in aureocin A53 and epidermicin NI01 gene clusters can be found. However, in the case of the latter, an additional ORF, with an unknown function, is present. Further downstream, in the same orientation as *bacSp222*, are genes encoding a putative membrane protein and an ABC transporter. Such transporters are also encoded within gene clusters of the other staphylococcal bacteriocins^19^,^21^,^30^. The genetic organization of the lacticin locus differs from those of BacSp222 and two others staphylococcal bacteriocins^24^. However, the presence of genes encoding ABC transporters and other membrane-associated proteins indicates their importance for the efficient production of leaderless bacteriocins.

The bactericidal activity of BacSp222 against various gram-positive cocci is evident, which may indicate its role in outcompeting other bacteria residing on the skin or mucosal surfaces. The MIC values for staphylococci were approximately 1 μM, which is a few times higher than those for epidermicin NI01 but slightly lower than the values for lacticin Q and lantibiotic BacCH91 from *S. aureus* CH91^20^,^21^,^22^,^30^. BacSp222 is lytic for susceptible bacteria because it induces efflux of intracellular content, as indicated by the efficient release of GFP from recombinant bacteria treated with the bacteriocin. Different modes of action for bactericidal peptides produced by bacteria, as well as eukaryotic organisms, have been proposed. Bacteriocins with a low molecular weight such as nisin, pediocin PA-1, and lacticin 3147 produced by lactic acid bacteria cause ion efflux via pore formation with the aid of initial receptors, known as docking molecules^18^. In turn, lacticin Q does not require a docking molecule and acts through a unique mechanism termed the “huge toroidal pore”^36^. The bactericidal activity of aureocin A53 is associated with generalized membrane destruction rather than with the formation of discrete pores^27^. To date, the mechanism of action of epidermicin NI01 has not been elucidated. However, the peptide has significant pharmacological potential due to its ability to prevent MRSA infections *in vivo* in insect models^21^. The observed protein (GFP) release upon BacSp222 treatment indicates massive disruption of cell membrane rather than subtle pore formation; however, the detailed mechanism of bacterial lysis needs to be further clarified experimentally.

The N-formylation of methionine in BacSp222 is unnecessary for bactericidal activity because the synthetic peptide lacking the modification exhibited even better activity against an indicator strain. The same effect has been observed for epidermicin NI01^21^. This result was somewhat expected because many bacteriocins are produced with leader peptides that are cleaved off during secretion, resulting in a free N-terminus. Moreover, leaderless lacticin Q produced *in vitro* using an insect cell extract, which presumably lacked N-terminal formylation, also retained its antimicrobial activity^24^. Because N-formylation of methionine is a consequence of the mode of protein synthesis in bacteria but not in eukaryotic organisms, the modification is of significance in bacterial interactions with the host. Indeed, N-formylated peptides (FP) are dominant neutrophil chemoattractants released by *S. aureus*^37^. The interaction of these peptides with host cells occurs through formyl peptide receptors (FPRs). FPR1 is the high-affinity receptor for FP that is activated by picomolar to nanomolar concentrations of FP, whereas FPR-like 1 (FPRL1, denoted also as FPR2/ALX) is considered a low-affinity receptor^15^,^38^. Importantly however, the latter senses phenol-soluble modulins (PSMs) produced in high quantities by community associated MRSA (CA-MRSA) and, through calcium ion influx and secretion of interleukin 8, initiates proinflammatory neutrophil responses to the pathogen^38^. BacSp222, however, did not activate the FPRL1 receptor overexpressed on recombinant CHO-K1 cells.

BacSp222 remains active upon even prolonged incubation at elevated temperature and is exceptionally resistant to proteases *in vitro.* The former feature is quite common for bacteriocins, which, in combination with the virtual lack of cytotoxicity, contributes to common and successful application of bacteriocins in the food industry as preservatives, either in the form of additives or as coating agents for packaging materials^39^,^40^. For the same reasons, bacteriocins are suggested to have great potential application in medicine and veterinary medicine as antiseptics or even drugs^41^. Resistance to proteases, although surprising for a linear nonmodified peptide, has also been reported for other staphylococcal bacteriocins, including aureocin A53 and BacCH91^19^,^20^. The stability of BacSp222 in the presence of biologically relevant proteases such as staphylococcal proteases and enzymes produced by neutrophils has been confirmed. However, in the case of epidermicin NI01, despite its high stability *in vitro*, the bacteriocin’s activity was lost within less than an hour after administration to *Galleria mellonella* larvae, indicating its degradation or inactivation by host agents^21^. Therefore, keeping in mind the cytotoxic and modulatory activity of BacSp222, the verification of the peptide’s performance using an *in vivo* model would be very intriguing. However, such studies are beyond the scope of the current research.

In addition to the evident bactericidal activity of BacSp222, the most intriguing findings are its cytotoxic and modulatory effects on mammalian cells. Generally, peptide bacteriocins are considered to be noncytotoxic^18^. However, the first known bacterial toxin active against eukaryotic and prokaryotic cells was a cytolysin produced by *Enterococcus faecalis*^42^. Moreover, adverse effects on mammalian cells have been reported for bovicin HC5 and nisin, albeit at high concentrations (tens to hundreds of micromoles/l)^43^. BacSp222 is cytotoxic at significantly lower doses (LD50 = 3.1 and 3.0 μM for HSF and keratinocytes, respectively). Slightly higher concentrations were needed for killing of two murine monocyte/macrophage cell lines, as well as HeLa cells and ASCs. Most likely, similarly to its bactericidal effects, the peptide’s cytotoxic mechanism is driven by the disruption of cell membrane integrity because the leakage of lactate dehydrogenase from the cells is a prerequisite for a positive LDH test. Moreover, the rapid influx of propidium iodide into cell nucleus was observed upon cell treatment with BacSp222. Keratinocytes and fibroblasts are the first cells to come in contact with *S. pseudintermedius* during colonization. Therefore, the high activity of BacSp222 towards these cells is probably of biological relevance. The bacteria may first successfully outcompete commensal bacterial inhabitants of the skin and mucosal surfaces by producing BacSp222 at concentrations up to a few micromolar. Then, the bacteria could turns to the newly colonized host by further producing the bacteriocin, which becomes toxic to epidermal cells that form the first line of defence against pathogens. However, as an opportunistic pathogen, *S. pseudintermedius* must precisely interact with the host to avoid premature clearance by the immune system. Therefore, the multifunctional BacSp222 may also modulate these interactions.

The peptide at nanomolar concentrations effectively enhances IFN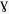-induced NO release in murine monocyte/macrophage cell lines P388D1 and RAW264.7. Nitric oxide acts as an essential messenger molecule in various inflammatory pathways^44^. The synthesis of NO by innate immune cells due to increased iNOS expression is also considered as an important host-defence mechanism against various pathogens (bacteria, parasites or viruses)^45^,^46^. However, a growing body of evidence indicates that NO acts as an immunosuppressor as well, mainly due to its ability to inhibit leukocyte infiltration, lymphocyte proliferation, cyclooxygenase activity, and cytokine expression^47^,^48^. Because the induction of iNOS expression is quite complex, only a few agents are able to stimulate NO production without costimulators. LPS is the most common stimulator of NO production by macrophages and is able to upregulate iNOS expression independently of other factors^44^. However, LPS is not produced by staphylococci, so the lack of increased NO production by BacSp222 in combination with LPS is understandable. In addition to LPS, antigens of *Streptococcus anginosus*^49^ and *Helicobacter pylori*^50^ are known inducers of NO synthesis. IFN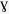 is an important stimulator and costimulator of iNOS expression. This cytokine is produced during infection and may enhance the innate immune response induced by many bacterial components by a mechanism involving increased expression of pattern recognition receptors (PRRs) on the cell surface, synthesis of IFN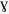 stimulated mediators, and sensitizing of many downstream signalling pathways^51^. Thus, the effect of BacSp222 on NO synthesis in cells incubated with IFN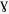 could be hypothesized to result from a priming of the activity of IFN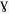, which then contributes to better responses to BacSp222.

In summary, we have demonstrated that BacSp222, in addition to exhibiting bactericidal activity, may affect homeostasis of the host through cytotoxic effects and by inducing or enhancing NO release by macrophages. Although the molecular mechanism and biological significance of this observation must be elucidated in further studies, BacSp222 could potentially be one of the first examples of multifunctional peptides that breaks the convention of splitting bacteriocins and virulence factors into two unrelated groups.

## Materials and Methods

### Bacterial strains and growth conditions

*S. pseudintermedius* strain 222 is a bacterium isolated from deep lesions in dog skin and was deposited in the collection of the Department of Microbiology, Faculty of Biochemistry, Biophysics and Biotechnology, Jagiellonian University (Krakow, Poland). Independently, the strain was also deposited under the accession number PCM 2791 in the Polish Collection of Microorganisms (Wroclaw, Poland). *Bacillus subtilis* ATCC 6633; *Lactococcus lactis* subsp. *lactis* LOCK 0871 strain 239; *Micrococcus luteus* ATCC 4698; *S. aureus* strains ATCC 25923, KB/8658 (a strain isolated from a dog), DSM 26258 (a bacteriocin-producing strain isolated from a chicken), and MRSA USA300 strain FPR3757; *S. epidermidis* ATCC 35547; *S. intermedius* ATCC 29663; *S. intermedius* R-2725 (a strain isolated from a dog); *S. pseudintermedius* LMG 22219; *S. saprophiticus* ATCC 15305; *Streptococcus pyogenes* PCM 465; *Streptococcus sanguinis* PCM 2335; *Escherichia coli* K12 ATCC 10798; *Klebsiella pneumoniae* ATCC 13886; *Serratia marcescens* ATCC 274; and *Candida albicans* ATCC 10231 were obtained from the collections of the Faculty of Biochemistry, Biophysics and Biotechnology, Jagiellonian University (Krakow, Poland). Gram-positive (except streptococci) and Gram-negative bacteria were cultivated at 37 °C in tryptic soy broth (TSB) and Luria-Bertani (LB) broth, respectively. Streptococci were grown on brain-heart infusion (BHI) agar (Sigma). *Candida* sp. was maintained in yeast extract-peptone-glucose (YPG) medium at 30 °C.

### Purification of BacSp222

Bacteriocin BacSp222 was purified from *S. pseudintermedius* 222 culture supernatant. The bacteria were cultivated in TSB at 37 °C and 200 rpm shaking for 18 h. The culture was centrifuged at 17 000 *g* for 15 min at 4 °C. The supernatant was cooled to 4 °C and precipitated with ammonium sulphate to 60% saturation. The precipitated material was recovered by centrifugation at 17 000 *g* for 30 min at 4 °C. The pellet was dissolved in 50% (v/v) acetonitrile, acidified to pH 3.0 with trifluoroacetic acid (TFA), centrifuged, and filtered through a 0.45 μm filter. The resulting solution was subjected to reverse-phase high pressure liquid chromatography (RP-HPLC) on a Nucleosil 300 C18 250 × 8 mm column (Macherey-Nagel) using a biphasic solvent system: A −0.1% TFA (v/v) in water and B −0.07% TFA in 80% acetonitrile (v/v). After equilibration of the column at 60% B, a linear gradient of 60 to 100% B was applied over 20 min using a flow rate of 1.5 ml/min, with spectrophotometric detection at 220 nm. The fraction containing BacSp222 was collected, dried in a centrifugal evaporator, dissolved in water acidified to pH 3.0 with TFA, and subjected to a second RP-HPLC chromatography step using a Kromasil C4 250 × 4.6 mm column (Sigma). The solvents described above were used, with a linear gradient of 68 to 75% B established over 20 min using a flow rate of 1 ml/min. The fraction containing BacSp222 was collected, dried in a centrifugal evaporator, and stored at −20 °C until further use.

### Protein chemistry techniques

The N-terminal amino acid sequence was determined in an automatic protein sequencer Procise 491 (Applied Biosystems) using Edman degradation of the polypeptide chain. Before sequencing, BacSp222 was deformylated by incubation for 24 h at room temperature in 0.6 M HCl, as described elsewhere. The concentration of purified BacSp222 was determined by amino acid analysis, as previously described^20^. In brief, the bacteriocin was hydrolysed in the gas phase in 6 M HCl at 115 °C for 24 h. The liberated amino acids were converted to phenylthiocarbamyl derivatives and analysed by HPLC (PicoTag 150 × 3.9 mm column; Waters). The total protein concentration was estimated using the bicinchoninic acid assay (BCA, Sigma). The concentration of BacSp222 in bacterial culture media was estimated by comparing the peptide peak height against a standard curve, which was prepared using injections of the culture medium enriched with known amounts of a BacSp222 standard. The estimations were performed by the direct injection of 20 μl of the clarified media on a Discovery BIO Wide Pore C5 250 × 4.6 mm column (Sigma). MALDI-ToF MS was carried out using an ultrafleXtreme mass spectrometer (Bruker). Tris-tricine sodium dodecyl sulphate polyacrylamide gel electrophoresis (SDS-PAGE) was carried out under reducing conditions using peptide-separating gels, according to the protocol of Schagger and von Jagow^52^. Following electrophoresis, the gels were fixed for 30 min in a mixture containing 50% methanol (v/v) and 10% acetic acid (v/v) and were then stained with Coomassie brilliant blue R-250. The BacSp222 without a formyl group at the N-terminus was obtained in a synthetic form from Tebu-Bio (France). The peptide deprived of N-terminal formyl-methionine was obtained by the overnight digestion of BacSp222 using cyanogen bromide in 70% (v/v) TFA. After digestion, the reaction mixture was dried in a centrifugal evaporator. Both peptides were further purified using the C4 column, as described above.

### Evaluation of the BacSp222 molecule hydrodynamic diameter

The hydrodynamic diameter of the BacSp222 molecule was determined at room temperature by the dynamic light scattering (DLS) technique using a Zetasizer Nano ZS apparatus (Malvern Instruments); a 0.2 mg/ml solution of the peptide in phosphate buffered saline (PBS) was used for analysis. Prior to measurements, the solution was filtered through a 0.2 μm bioinert membrane (Sigma). The obtained spectra were averaged over three independent measurements. The size of BacSp222 was also evaluated by gel filtration chromatography using a Superdex Peptide 10/300 GL column (GH Healthcare) operated in PBS with a 0.3 ml/min flow rate.

### Circular dichroism

The circular dichroism (CD) spectra of BacSp222 (125 μg/ml in PBS) were recorded using a J-715 spectropolarimeter (Jasco). The spectra were recorded at room temperature in the range 190–240 nm and were averaged over three independent passes. The amount of secondary structures was estimated by fitting the measured spectra to the reference spectra of natural native proteins (Prot, Jasco) or synthetic polypeptides (Yang, Jasco). To evaluate the conformational stability of the BacSp222 molecule at different temperatures, the ellipticity of the polarized light was recorded at 220 nm over the temperature range 20–90 °C at 1 °C/min increments.

### Quantitative reverse transcription PCR (RT-qPCR)

To collect RNA, bacteria were harvested from liquid culture by centrifugation at different time points. The cells were mechanically disrupted in TRI Reagent (Sigma) using glass beads. The subsequent steps were performed using a GeneJET RNA Purification Kit (Thermo Scientific). DNA contamination was removed with an On-Column DNase I Digestion Set (Sigma). The reverse transcription reaction was carried out according to the manufacturer’s protocol using RevertAid Premium Reverse Transcriptase (Thermo Scientific), 2 μg of total RNA as the template, and random hexanucleotides (Genomed). The qPCR reaction mixture was composed of 5 μl of the reverse transcription samples diluted 100-fold in water, 0.75 μl of a pair of gene-specific primers (10 μM each; Supplementary Materials Table S1), 1 μl of nuclease-free water, and 7.5 μl of iTaq Universal SYBR Green Super Mix (Bio-Rad). The reactions were carried out in a CFX96 Touch™ Real-Time PCR Detection System (Bio-Rad) according to the following program: 1. 95 °C 2 min; 2. 95 °C 30 s; 3. 55 °C 30 s (fluorescence measurement); 4. 74 °C 30 s; 5. repeat steps 2–4 40 times; melting curve analysis from 65 to 95 °C with a 0.5 °C increase step. The data obtained were analysed using the ΔΔCt method^53^. The 23S rRNA was used as a reference.

### Plasmid sequencing

The sequence of the p222 plasmid was obtained through a process of *de novo S. pseudintermedius* 222 whole genome assembly using MIRA software and MiSeq (Illumina) sequencing results. The plasmid sequence was obtained as one high-quality contig, as assessed using Gap5 software^54^. The semiautomated analysis of possible open reading frames and their homology to existing ones was performed using the BioPython^55^ library and its modules for NCBI Entrez databases and NCBI, as well as standalone BLAST tools^56^. The plasmid sequence has been deposited under the accession number CP011490 (GenBank).

### Determination of MIC values

The microbicidal activity of BacSp222 was determined using the microdilution method in compliance with Clinical and Laboratory Standards Institute (CLSI, for bacteria) guidelines M7-A7^57^ and in compliance with the European Committee on Antimicrobial Susceptibility Testing - Subcommittee on Antifungal Susceptibility Testing (EUCAST-AFST, for fungi)^58^. Briefly, the microorganisms were cultivated in dedicated liquid media to reach the early exponential phase or, in the case of *Streptococcus* spp., grown overnight on agar MHB supplemented with 2.5% (v/v) lysed horse blood (LHB). Subsequently, the microorganisms were diluted to 1 × 10^6^ CFU/ml (estimated using an Eddy Jet 2 automatic spiral plater, IUL) in MHB; in MHB supplemented with 5% (v/v) LHB (for streptococci); or, in the case of *Candida albicans*, in RPMI-1640 medium (Thermo) without L-Glu and phenol red, supplemented with 0.6 g/l L-Glu, 34.53 g/l 3-(N-morpholino)propanesulphonic acid and 18 g/l glucose, pH 7.0. Fifty microliters of microorganism suspension were mixed with an equal volume of appropriate medium containing serial 2- or 1.5-fold dilutions of BacSp222. After the cultures were incubated overnight at 37 °C, the optical density (OD) at 600 nm was measured. The minimal inhibitory concentration (MIC), calculated as a mean value from three independent measurements, was defined as the minimum peptide concentration for which an increase in OD was not observed compared to uninoculated broth.

### Radial diffusion assay

The bactericidal activity of BacSp222 was also determined using the radial diffusion assay. In TSB agar (1%, w/v) containing a 200× diluted overnight culture of *Bacillus subtilis* ATCC 6633, wells of 2.4-mm diameter were created by drilling out the agar and filling the hole with 10 μl of the BacSp222 preparation for testing. Following overnight incubation at 37 °C, the bactericidal activity was measured as the area of the inhibition zones.

### Green fluorescent protein (GFP) release assay

The bacteria transformed with GFP-encoding plasmids (pCN68^59^ for *S. aureus* RN4220; pALCP2G^60^ for *B. subtilis* 1A751^61^ and *E. coli* DH5α) were grown overnight in TSB supplemented with appropriate antibiotics, washed twice and finally suspended in PBS. Fifty microliters of the suspensions containing 2 × 10^6^ CFU of bacteria were then incubated for 1 h at 37 °C with serial dilutions of BacSp222 at concentrations ranging from 0.23 to 15 μM. Then, the bacteria were spun down, and the fluorescence in the supernatant was determined (Synergy H1 microplate reader, BioTek, 480/510 nm excitation/emission wavelengths). As the controls for 100% GFP release, *S. aureus* and *B. subtilis* were lysed using lysostaphin and chicken egg white lysozyme (Sigma), respectively, whereas the *E. coli* cells were disrupted by ultrasonication.

### Influence of temperature and proteolytic enzymes on BacSp222 activity

The temperature stability of BacSp222 (30 μM final concentration) was determined in PBS. The solution was incubated for 1 or 3 h at 72 °C (typical temperature of pasteurization) or at 100 °C. Independently, a solution of bacteriocin was also subjected to a full cycle of autoclaving (20 min at 121 °C). The susceptibility of BacSp222 to proteolysis was verified using *S. aureus* staphopain C and V8 peptidase, human neutrophil elastase, human cathepsin G (Biocentrum), bovine TPCK-treated trypsin, pepsin from porcine gastric mucosa (Sigma), and proteinase K (A&A Biotechnology). The dried samples of BacSp222 were dissolved to a final concentration of 30 μM in the following buffers: 100 mM Tris-HCl pH 8.0 containing 5 mM CaCl_2_ (for trypsin and proteinase K), 100 mM Tris-HCl pH 7.5 containing 5 mM cysteine hydrochloride and 5 mM EDTA (for staphopain C), 100 mM Tris-HCl pH 8.0 (for elastase), 100 mM Tris-HCl pH 7.5 containing 500 mM NaCl (for cathepsin G), 100 mM sodium phosphate pH 7.8 (for V8 peptidase), and 100 mM ammonium acetate pH 4.0 (for pepsin). The peptidases were added at 1:50 or 1:10 (enzyme:BacSp222) ratios by weight, and the mixtures were incubated for 1 or 3 h at 37 °C. The residual activity was determined with the radial diffusion assay using *Bacillus subtilis* as an indicator strain. All of the above experiments were performed in triplicate.

### Cytotoxicity and haemolytic assays

Human skin fibroblasts (HSF, ATCC CRL-2522), adipose-derived stem cells (ASCs, ATCC PCS-500-011), and HeLa human epithelioid cervix carcinoma cells (ATCC CCL-2) were maintained in Dulbecco’s modified Eagle’s medium (DMEM, Sigma) containing 1 g/l glucose, 10% (v/v) foetal bovine serum (FBS), 1 U/ml penicillin, 1 μg/ml streptomycin, and 2.5 μg/ml amphotericin B. Murine P388/D1 monocyte/macrophage cells (ATCC CCL-46) and murine RAW264.7 macrophage-like cells (ATCC TIB-71) were cultured in DMEM containing 4.5 g/l glucose, 5% (v/v) FBS, and antibiotics, as indicated above. Keratinocytes from human skin were grown in KBM Gold Medium (Lonza). All cells were incubated in standard conditions (5% CO_2_, 37 °C, >95% humidity). For the viability and cytotoxicity tests, the cells were transferred into a 96-well plate at a density of 2 × 10^5^ cells per well. After 24 h, the medium was replaced with 50 μl of fresh medium containing 5% (v/v) FBS (HSF, ASC, HeLa, and RAW cells) or of KBM Gold medium (keratinocytes), all with BacSp222 at different concentrations. Before use, the peptide solution was tested for lipopolysaccharide (LPS) contamination using an E-TOXATE assay kit (Sigma). The cells were then exposed to BacSp222 for 4 h and then subjected to the lactate dehydrogenase (LDH) and the tetrazolium salt reduction (MTT) assays. LDH release was performed according to the protocol provided by the manufacturer (CytoTox 96 kit, Promega, USA). Briefly, 30 μl of cultured media was transferred into a microplate and mixed with 30 μl of an INT substrate. After a 30-min incubation (RT, in the dark), the reaction was stopped, and the absorbance was measured with a microplate reader (PowerWave X, BioTek Instruments) at 490 nm, followed by subtraction of the absorbance reference value at 690 nm. The obtained results were normalized to the value of the total cellular LDH activity (positive control: cells lysed with 1% (v/v) Triton X-100, reflecting maximal LDH release from the cells). For the MTT assay, the cells were incubated with 55 μl of fresh DMEM containing MTT (0.5 mg/ml) for 3 h at 37 °C, and then the media was replaced with 100 μl acidified isopropanol to dissolve the water-insoluble formazan crystals produced in living cells with maintained reductive potential. The absorbance of solubilized purple formazan was measured at 540 nm, followed by subtraction of the absorbance reference value at 690 nm. The obtained results were normalized to the value of the control without BacSp222. To track cell membrane integrity, HSF (2 × 10^4^ cells per well) were incubated in DMEM without phenol red containing 10% (v/v) FBS, 0.5, 15 μM BacSp222, 1 ng/ml propidium iodide (PI, Sigma) and 10 ng/ml fluorescein diacetate (FDA, Sigma). The cells were observed using an Eclipse Ti (Nikon) microscope at 488/515–550 nm (FDA) or 540/605–660 nm (PI) excitation/emission wavelengths, respectively. To evaluate haemolytic activity, a suspension of 3% (v/v) human erythrocytes in PBS was incubated for 1 h with various amounts of BacSp222. The amount of released haemoglobin was spectrophotometrically determined at 540 nm and compared with a positive control (erythrocytes lysed with 1% sodium dodecyl sulphate).

### Analysis of the influence of BacSp222 on iNOS production

The murine monocyte/macrophage cell line P388/D1 and murine macrophage-like cell line RAW264.7 were grown in a 96-well plate in DMEM supplemented with 5% (v/v) FBS. Prior to the experiment, the medium was replaced with fresh DMEM enriched with 2% (v/v) of FBS (negative control) or supplemented with 1) 100 ng/ml lipopolysaccharide (LPS, Sigma), 2) 10 ng/ml IFN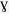 (R&D Systems), 3) 0.01–1000 nM BacSp222, or 4) BacSp222 combined with LPS or IFN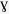. The cells were then cultured for another 24 h to induce iNOS expression and nitric oxide synthesis. The nitrite levels were measured in culture medium by a nitrate assay performed on a microplate. One hundred microliters of cultured media was incubated with 100 μl of Griess reagent (1% (w/v) sulphanilic acid/0.1% (w/v) N-(1-naphtyl) ethylenediaminedihydrochloride (Sigma) in 2.5% (v/v) H_3_PO_4_) at room temperature for 10 min. Then, the absorbance was measured at 545 nm using a microplate reader (PowerWave X, BioTek Instruments). Before use, the solution of BacSp222 was tested for LPS contamination using an E-TOXATE assay kit (Sigma). The measurements ascertained that the level of LPS in 1 μM bacteriocin solution was equal to or less than 3 pg/ml. As verified in a control experiment (data not shown), such a concentration did not influenced iNOS activity in the cells used.

### Formyl peptide receptor assay

Activation of the cellular formyl-peptide receptor was assayed using cAMPZen FroZen N-formyl peptide (FPRL1) human recombinant CHO-K1 cells (Perkin-Elmer) and a cAMP-GLO bioluminescent assay (Promega) largely according to the protocols provided by the manufacturers. Formyl-methionyl-leucyl-phenylalanine peptide (fMLP, Sigma) was used as a positive control, and the BacSp222 concentrations tested were 0.01, 0.1, 1.0, 10, 100, 1000, and 5000 nM.

## Additional Information

**How to cite this article**: Wladyka, B. *et al.* A peptide factor secreted by *Staphylococcus pseudintermedius* exhibits properties of both bacteriocins and virulence factors. *Sci. Rep.* **5**, 14569; doi: 10.1038/srep14569 (2015).

## Supplementary Material

Supplementary Information

## Figures and Tables

**Table 1:** MIC values of BacSp222 toward different microorganisms.

Microorganism	MIC ± SD (μM)
*Bacillus subtilis* ATCC 6633	0.16 ± 0.00
*Lactococcus lactis* subsp. *lactis* ŁOCK 0871 strain 239	0.89 ± 0.00
*Micrococcus luteus* ATCC 4698	0.11 ± 0.07
*Staphylococcus aureus* DSM 26258 (CH91)	0.92 ± 0.23
*Staphylococcus aureus* MRSA USA300 strain FPR3757	0.89 ± 0.00
*Staphylococcus aureus* KB/8658	1.30 ± 0.00
*Staphylococcus aureus* ATCC 25923	1.00 ± 0.36
*Staphylococcus epidermidis* ATCC 35547	4.40 ± 0.00
*Staphylococcus intermedius* ATCC 29663	2.00±0.00
*Staphylococcus intermedius* R-2725	1.20 ± 0.24
*Staphylococcus pseudintermedius* 222	2.10 ± 0.46
*Staphylococcus pseudintermedius* LMG 22219	0.16 ± 0.00
*Staphylococcus saprophyticus* ATCC 15305	0.93 ± 0.36
*Streptococcus pyogenes* PCM 465	7.80 ± 1.92
*Streptococcus sanguinis* PCM 2335	3.50 ± 0.85
*Escherichia coli* K12	>100
*Klebsiella pneumoniae* ATCC 13886	>100
*Serratia marcescens* ATCC 274	>100
*Candida albicans* ATCC 10231	100 ± 0.00

## References

[b1] Weese J. S. & van Duijkeren E. Methicillin-resistant *Staphylococcus aureus* and *Staphylococcus pseudintermedius* in veterinary medicine. Vet Microbiol 140, 418–429 (2010). 19246166 10.1016/j.vetmic.2009.01.039

[b2] Van Hoovels L., Vankeerberghen A., Boel A., Van Vaerenbergh K. & De Beenhouwer H. First case of *Staphylococcus pseudintermedius* infection in a human. J Clin Microbiol 44, 4609–4612 (2006). 17050817 10.1128/JCM.01308-06 PMC1698428

[b3] Ruscher C. *et al.* Prevalence of Methicillin-resistant *Staphylococcus pseudintermedius* isolated from clinical samples of companion animals and equidaes. Vet Microbiol 136, 197–201 (2009). 19097710 10.1016/j.vetmic.2008.10.023

[b4] Savini V. *et al.* Methicillin-resistant *Staphylococcus pseudintermedius* infection in a bone marrow transplant recipient. J Clin Microbiol 51, 1636–1638 (2013). 23486715 10.1128/JCM.03310-12 PMC3647946

[b5] Starlander G., Borjesson S., Gronlund-Andersson U., Tellgren-Roth C. & Melhus A. Cluster of infections caused by methicillin-resistant *Staphylococcus pseudintermedius* in humans in a tertiary hospital. J Clin Microbiol 52, 3118–3120 (2014). 24871217 10.1128/JCM.00703-14 PMC4136194

[b6] Bukowski M. *et al.* Species determination within *Staphylococcus* genus by extended PCR-restriction fragment length polymorphism of saoC gene. FEMS Microbiol Lett 362, 1–11 (2015). 25790489 10.1093/femsle/fnu007

[b7] Sasaki T. *et al.* Reclassification of phenotypically identified *Staphylococcus intermedius* strains. J Clin Microbiol 45, 2770–2778 (2007). 17596353 10.1128/JCM.00360-07 PMC2045239

[b8] Borjesson S., Gomez-Sanz E., Ekstrom K., Torres C. & Gronlund U. *Staphylococcus pseudintermedius* can be misdiagnosed as *Staphylococcus aureus* in humans with dog bite wounds. Eur J Clin Microbiol Infect Dis 34, 839–844 (2015). 25532507 10.1007/s10096-014-2300-y

[b9] van Duijkeren E. *et al.* Review on methicillin-resistant *Staphylococcus pseudintermedius*. J Antimicrob Chemother 66, 2705–2714 (2011). 21930571 10.1093/jac/dkr367

[b10] Ben Zakour N. L., Beatson S. A., van den Broek A. H., Thoday K. L. & Fitzgerald J. R. Comparative genomics of the *Staphylococcus intermedius* group of animal pathogens. Front Cell Infect Microbiol 2, 44 (2012). 22919635 10.3389/fcimb.2012.00044 PMC3417630

[b11] Youn J. H., Moodley A., Park Y. H. & Sugimoto C. Genome Sequence of Methicillin-Resistant *Staphylococcus pseudintermedius* Sequence Type 233 (ST233) Strain K7, of Human Origin. Genome Announc 1 (2013). 10.1128/genomeA.00310-13 PMC3707588 23788539

[b12] Geoghegan J. A., Smith E. J., Speziale P. & Foster T. J. *Staphylococcus pseudintermedius* expresses surface proteins that closely resemble those from Staphylococcus aureus. Vet Microbiol 138, 345–352 (2009). 19372010 10.1016/j.vetmic.2009.03.030

[b13] Foster T. J. Colonization and infection of the human host by staphylococci: adhesion, survival and immune evasion. Vet Dermatol 20, 456–470 (2009). 20178484 10.1111/j.1365-3164.2009.00825.x

[b14] Dubin G., Koziel J., Pyrc K., Wladyka B. & Potempa J. Bacterial proteases in disease - role in intracellular survival, evasion of coagulation/ fibrinolysis innate defenses, toxicoses and viral infections. Curr Pharm Des 19, 1090–1113 (2013). 23016681 10.2174/1381612811319060011

[b15] Prat C., Bestebroer J., de Haas C. J., van Strijp J. A. & van Kessel K. P. A new staphylococcal anti-inflammatory protein that antagonizes the formyl peptide receptor-like 1. J Immunol 177, 8017–8026 (2006). 17114475 10.4049/jimmunol.177.11.8017

[b16] de Haas C. J. *et al.* Chemotaxis inhibitory protein of *Staphylococcus aureus*, a bacterial antiinflammatory agent. J Exp Med 199, 687–695 (2004). 14993252 10.1084/jem.20031636 PMC2213298

[b17] Grumann D., Nubel U. & Broker B. M. *Staphylococcus aureus* toxins–their functions and genetics. Infect Genet Evol 21, 583–592 (2014). 23541411 10.1016/j.meegid.2013.03.013

[b18] Riley M. A. & Wertz J. E. Bacteriocins: evolution, ecology, and application. Annu Rev Microbiol 56, 117–137 (2002). 12142491 10.1146/annurev.micro.56.012302.161024

[b19] Netz D. J. *et al.* Biochemical characterisation and genetic analysis of aureocin A53, a new, atypical bacteriocin from *Staphylococcus aureus*. J Mol Biol 319, 745–756 (2002). 12054867 10.1016/S0022-2836(02)00368-6

[b20] Wladyka B. *et al.* Isolation, biochemical characterization, and cloning of a bacteriocin from the poultry-associated *Staphylococcus aureus* strain CH-91. Appl Microbiol Biotechnol 97, 7229–7239 (2013). 23196985 10.1007/s00253-012-4578-y PMC3724985

[b21] Gibreel T. M. & Upton M. Synthetic epidermicin NI01 can protect *Galleria mellonella* larvae from infection with *Staphylococcus aureus*. J Antimicrob Chemother 68, 2269–2273 (2013). 23711896 10.1093/jac/dkt195

[b22] Fujita K. *et al.* Structural analysis and characterization of lacticin Q, a novel bacteriocin belonging to a new family of unmodified bacteriocins of gram-positive bacteria. Appl Environ Microbiol 73, 2871–2877 (2007). 17351096 10.1128/AEM.02286-06 PMC1892864

[b23] Iwatani S., Zendo T., Yoneyama F., Nakayama J. & Sonomoto K. Characterization and structure analysis of a novel bacteriocin, lacticin Z, produced by *Lactococcus lactis* QU 14. Biosci Biotechnol Biochem 71, 1984–1992 (2007). 17690480 10.1271/bbb.70169

[b24] Iwatani S. *et al.* Identification of the genes involved in the secretion and self-immunity of lacticin Q, an unmodified leaderless bacteriocin from *Lactococcus lactis* QU 5. Microbiology 158, 2927–2935 (2012). 23103973 10.1099/mic.0.062943-0

[b25] Yoneyama F. *et al.* Lacticin Q-mediated selective toxicity depending on physicochemical features of membrane components. Antimicrob Agents Chemother 55, 2446–2450 (2011). 21282423 10.1128/AAC.00808-10 PMC3088220

[b26] Nascimento Jdos S. *et al.* Genes involved in immunity to and secretion of aureocin A53, an atypical class II bacteriocin produced by *Staphylococcus aureus* A53. J Bacteriol 194, 875–883 (2012). 22155775 10.1128/JB.06203-11 PMC3272949

[b27] Netz D. J., Bastos Mdo C. & Sahl H. G. Mode of action of the antimicrobial peptide aureocin A53 from *Staphylococcus aureus*. Appl Environ Microbiol 68, 5274–5280 (2002). 12406714 10.1128/AEM.68.11.5274-5280.2002 PMC129900

[b28] Takeuchi K., Nakatani Y. & Hisatomi O. Accuracy of protein size estimates based on light scattering measurements. Open Journal of Biophysics 4, 83–91 (2014).

[b29] Butcher B. G. & Helmann J. D. Identification of *Bacillus subtilis* sigma-dependent genes that provide intrinsic resistance to antimicrobial compounds produced by Bacilli. Mol Microbiol 60, 765–782 (2006). 16629676 10.1111/j.1365-2958.2006.05131.x

[b30] Sandiford S. & Upton M. Identification, characterization, and recombinant expression of epidermicin NI01, a novel unmodified bacteriocin produced by *Staphylococcus epidermidis* that displays potent activity against Staphylococci. Antimicrob Agents Chemother 56, 1539–1547 (2012). 22155816 10.1128/AAC.05397-11 PMC3294953

[b31] Bae Y. S. *et al.* Identification of peptides that antagonize formyl peptide receptor-like 1-mediated signaling. J Immunol 173, 607–614 (2004). 15210823 10.4049/jimmunol.173.1.607

[b32] Prat C. *et al.* A homolog of formyl peptide receptor-like 1 (FPRL1) inhibitor from *Staphylococcus aureus* (FPRL1 inhibitory protein) that inhibits FPRL1 and FPR. J Immunol 183, 6569–6578 (2009). 19846866 10.4049/jimmunol.0801523

[b33] Lautz S. *et al.* Dissemination of the gene encoding exfoliative toxin of *Staphylococcus intermedius* among strains isolated from dogs during routine microbiological diagnostics. J Vet Med B Infect Dis Vet Public Health 53, 434–438 (2006). 17062121 10.1111/j.1439-0450.2006.00999.x

[b34] Dufour P. *et al.* High genetic variability of the agr locus in *Staphylococcus* species. J Bacteriol 184, 1180–1186 (2002). 11807079 10.1128/jb.184.4.1180-1186.2002 PMC134794

[b35] Pinto T. S. *et al.* Evidence for production of a bacteriocin-like substance by *Staphylococcus pseudintermedius*, inhibitory to Staphylococcus aureus from foods. Nat Prod Res 27, 1098–1101 (2013). 22703567 10.1080/14786419.2012.696260

[b36] Yoneyama F. *et al.* Peptide-lipid huge toroidal pore, a new antimicrobial mechanism mediated by a lactococcal bacteriocin, lacticin Q. Antimicrob Agents Chemother 53, 3211–3217 (2009). 19470516 10.1128/AAC.00209-09 PMC2715615

[b37] Durr M. C. *et al.* Neutrophil chemotaxis by pathogen-associated molecular patterns–formylated peptides are crucial but not the sole neutrophil attractants produced by *Staphylococcus aureus*. Cell Microbiol 8, 207–217 (2006). 16441432 10.1111/j.1462-5822.2005.00610.x

[b38] Kretschmer D. *et al.* Human formyl peptide receptor 2 senses highly pathogenic *Staphylococcus aureus*. Cell Host Microbe 7, 463–473 (2010). 20542250 10.1016/j.chom.2010.05.012 PMC3417054

[b39] La Storia A., Ercolini D., Marinello F. & Mauriello G. Characterization of bacteriocin-coated antimicrobial polyethylene films by atomic force microscopy. J Food Sci 73, T48–54 (2008). 18460145 10.1111/j.1750-3841.2008.00713.x

[b40] Mauriello G., Ercolini D., La Storia A., Casaburi A. & Villani F. Development of polythene films for food packaging activated with an antilisterial bacteriocin from *Lactobacillus curvatus* 32Y. J Appl Microbiol 97, 314–322 (2004). 15239697 10.1111/j.1365-2672.2004.02299.x

[b41] Bastos M. C., Ceotto H., Coelho M. L. & Nascimento J. S. Staphylococcal antimicrobial peptides: relevant properties and potential biotechnological applications. Curr Pharm Biotechnol 10, 38–61 (2009). 19149589 10.2174/138920109787048580

[b42] Coburn P. S. & Gilmore M. S. The *Enterococcus faecalis* cytolysin: a novel toxin active against eukaryotic and prokaryotic cells. Cell Microbiol 5, 661–669 (2003). 12969372 10.1046/j.1462-5822.2003.00310.x

[b43] Paiva A. D. *et al.* Toxicity of bovicin HC5 against mammalian cell lines and the role of cholesterol in bacteriocin activity. Microbiology-Sgm 158, 2851–2858 (2012). 10.1099/mic.0.062190-0 22956757

[b44] Nussler A. K. & Billiar T. R. Inflammation, immunoregulation, and inducible nitric-oxide synthase. Journal of Leukocyte Biology 54, 171–178 (1993). 7689630

[b45] Pacelli R. *et al.* Nitric-oxide potentiates hydrogen peroxide-induced killing of *Escherichia coli*. Journal of Experimental Medicine 182, 1469–1479 (1995). 7595217 10.1084/jem.182.5.1469 PMC2192188

[b46] Flesch I. E. A. & Kaufmann S. H. E. Mechanisms involved in mycobacterial growth-inhibition by gamma interferon-activated bone-marrow macrophages - role of reactive nitrogen intermediates. Infection and Immunity 59, 3213–3218 (1991). 1908829 10.1128/iai.59.9.3213-3218.1991 PMC258155

[b47] Mills C. D. Molecular-basis of suppressor macrophages - arginine metabolism via the nitric-oxide synthetase pathway. Journal of Immunology 146, 2719–2723 (1991). 1707918

[b48] Stadler J. *et al.* Endogenous nitric oxide inhibits the synthesis of cyclooxygenase products and interleukin-6 by rat Kupffer cells. J Leukoc Biol 53, 165–172 (1993). 8445328 10.1002/jlb.53.2.165

[b49] Sasaki M. *et al.* Antigenic characterisation of a novel *Streptococcus anginosus* antigen that induces nitric oxide synthesis by murine peritoneal exudate cells. Journal of Medical Microbiology 50, 952–958 (2001). 11699591 10.1099/0022-1317-50-11-952

[b50] Assmann I. A. *et al.* Role of virulence factors, cell components and adhesion in *Helicobacter pylori*-mediated iNOS induction in murine macrophages. Fems Immunology and Medical Microbiology 30, 133–138 (2001). 11267846 10.1111/j.1574-695X.2001.tb01561.x

[b51] Held T. K., Xiao W. H., Liang Y., Kalvakolanu D. V. & Cross A. S. Gamma interferon augments macrophage activation by lipopolysaccharide by two distinct mechanisms, at the signal transduction level and via an autocrine mechanism involving tumor necrosis factor alpha and interleukin-1. Infection and Immunity 67, 206–212 (1999). 9864217 10.1128/iai.67.1.206-212.1999 PMC96298

[b52] Schagger H. & von Jagow G. Tricine-sodium dodecyl sulfate-polyacrylamide gel electrophoresis for the separation of proteins in the range from 1 to 100 kDa. Anal Biochem 166, 368–379 (1987). 2449095 10.1016/0003-2697(87)90587-2

[b53] Livak K. J. & Schmittgen T. D. Analysis of relative gene expression data using real-time quantitative PCR and the 2(-Delta Delta C(T)) Method. Methods 25, 402–408 (2001). 11846609 10.1006/meth.2001.1262

[b54] Bonfield J. K. & Whitwham A. Gap5–editing the billion fragment sequence assembly. Bioinformatics 26, 1699–1703 (2010). 20513662 10.1093/bioinformatics/btq268 PMC2894512

[b55] Cock P. J. *et al.* Biopython: freely available Python tools for computational molecular biology and bioinformatics. Bioinformatics 25, 1422–1423 (2009). 19304878 10.1093/bioinformatics/btp163 PMC2682512

[b56] Camacho C. *et al.* BLAST+: architecture and applications. BMC Bioinformatics 10, 421 (2009). 20003500 10.1186/1471-2105-10-421 PMC2803857

[b57] CLSI - Clinical and Laboratory Standards Institute. M07-A9. Methods for dilution antimicrobial susceptibility tests for bacteria that grow aerobically; approved standard. Ninth edition (Clinical and Laboratory Standards Institute, 2012).

[b58] Arendrup M. C., Cuenca-Estrella M., Lass-Florl C. & Hope W. EUCAST technical note on the EUCAST definitive document EDef 7.2: method for the determination of broth dilution minimum inhibitory concentrations of antifungal agents for yeasts EDef 7.2 (EUCAST-AFST). Clin Microbiol Infect 18, E246–247 (2012). 22563750 10.1111/j.1469-0691.2012.03880.x

[b59] Charpentier E. *et al.* Novel cassette-based shuttle vector system for gram-positive bacteria. Appl Environ Microbiol 70, 6076–6085 (2004). 15466553 10.1128/AEM.70.10.6076-6085.2004 PMC522135

[b60] Bukowski M. *et al.* A regulatory role for *Staphylococcus aureus* toxin-antitoxin system PemIKSa. Nat Commun 4, 2012 (2013). 23774061 10.1038/ncomms3012

[b61] Lam K. H., Chow K. C. & Wong W. K. Construction of an efficient *Bacillus subtilis* system for extracellular production of heterologous proteins. J Biotechnol 63, 167–177 (1998). 9803531 10.1016/s0168-1656(98)00041-8

