# Supplementary material for: A peptide factor secreted by Staphylococcus pseudintermedius exhibits properties of both bacteriocins and virulence factors
Source: Sci Rep. 2015 Sep 28;5:14569. doi: 10.1038/srep14569 (PMC4585962; doi:10.1038/srep14569)
Supplement: Supplementary Information [file srep14569-s1.doc]

**Supplementary Materials**

**A peptide factor secreted by *Staphylococcus pseudintermedius* exhibits properties of both bacteriocins and virulence factors**

**Benedykt Wladyka, Marcin Piejko, Monika Bzowska, Piotr Pieta, Monika Krzysik, Łukasz Mazurek, Ibeth Guevara-Lora, Michał Bukowski, Artur J. Sabat, Alexander W. Friedrich, Emilia Bonar, Jacek Międzobrodzki, Adam Dubin, Paweł Mak**

**Supplementary Table S1. Sequences of primers used in the study.**

| Primer | Sequence (5’-3’) |
| --- | --- |
| BacSp222_qPCR_F | GTCGCGCCTTATACAATTGGG |
| BacSp222_qPCR_R | GTAGCTCCTGATTTAAGCCACTCC |
| 23SrRNA_F | CCGAAGCTGTGGATTGTCCT |
| 23SrRNA_R | GCTACTCACACCGGCATTCT |

**Supplementary Table S2. Purification table of BacSp222.**

| Step | Total volume (ml) | Total protein (mg) | Total activity  (U)* | Specific activity (U/mg) | Degree of purification (fold) | Yield (%) |
| --- | --- | --- | --- | --- | --- | --- |
| Culture medium | 860.0 | 1565.2 | 17200 | 10.9 | 1.0 | 100 |
| Ammonium sulfate precipitation | 3.7 | 27.1 | 6166 | 227.4 | 20.0 | 36 |
| C18 column | 1.8 | 5.5 | 1500 | 272.2 | 24.9 | 9 |
| C4 column (final preparation) | 0.5 | 0.7 | 833 | 1156.9 | 105.4 | 5 |

*One unit (U) was defined as the activity of the peptide resulting in a growth inhibition zone of a 12-mm diameter, as determined in the radial diffusion assay using *Bacillus subtilis* ATCC 6633 as an indicator strain.

**B**


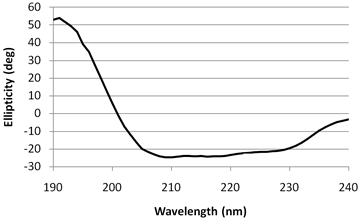

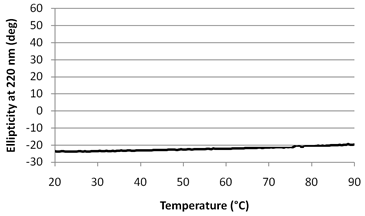


**A**

**Supplementary Figure S1.** **Circular dichroism studies of BacSp222.** (A) The spectrum of the peptide solution in PBS; (B) the elipticity of the peptide solution at 220 nm at different temperatures.


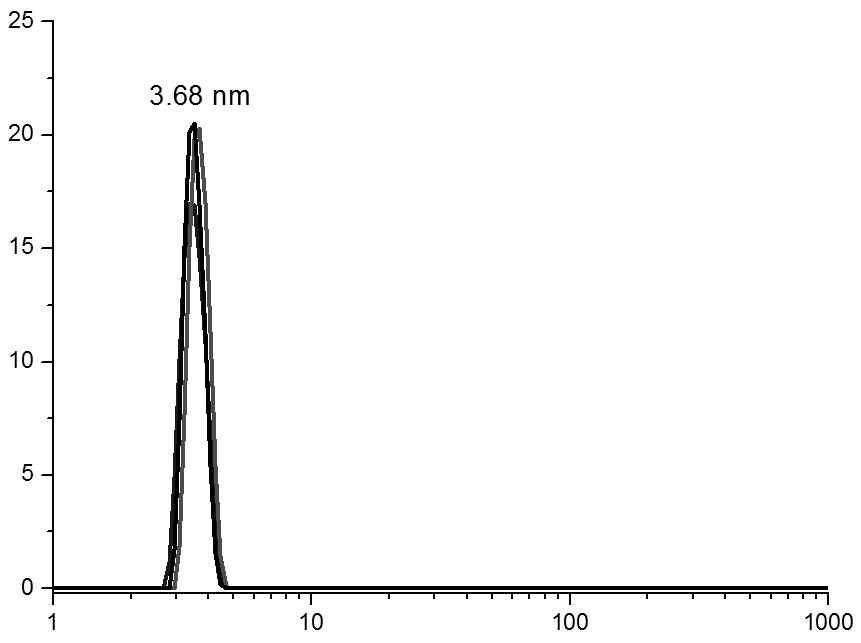


Diameter (nm)

Volume (%)

**Supplementary Figure S2.** **The DLS spectrum of BacSp222.**


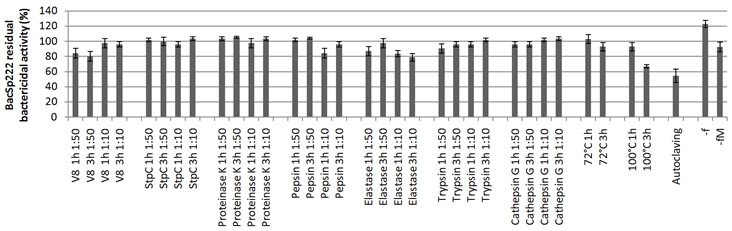


**Supplementary Figure S3.** **The residual bactericidal activity of BacSp222.** The activity of the peptide was assessed after digestion by peptidases, exposure to elevated temperatures, or removal of the N-terminal formyl group (-f), as well as N-terminal formyl methionine (-fM). The enzymatic digestion was conducted for 1 or 3 h at enzyme:peptide ratios of 1:50 and 1:10 (w/w). 100% bactericidal activity refers to the activity of untreated and unmodified BacSp222. The bars represent the average values from three independent experiments ± SD.


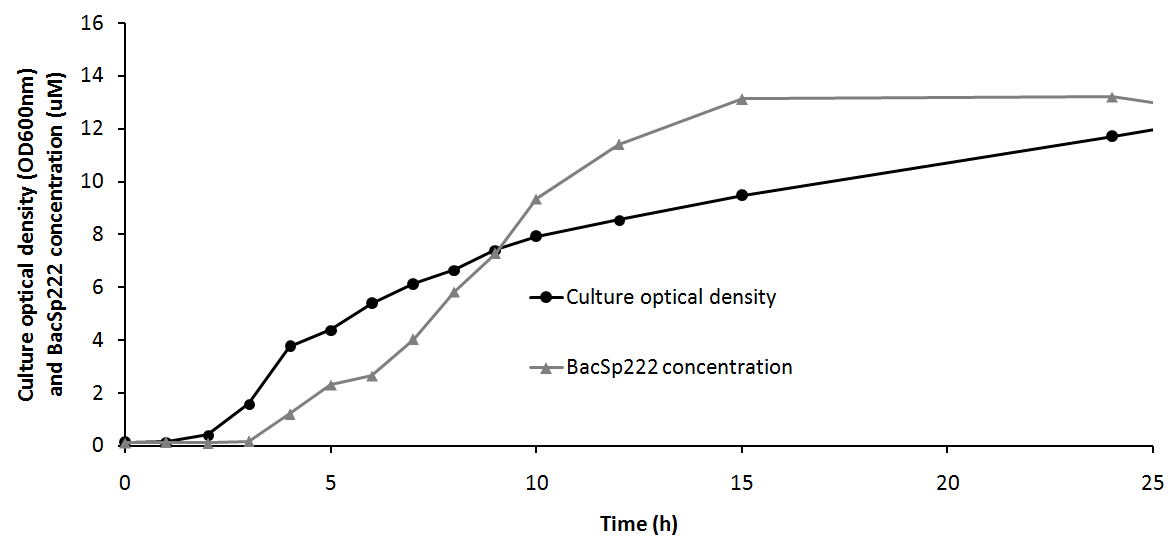


**Supplementary Figure S4.** **Optical density of and BacSp222 levels in the growth medium during *Staphylococcus pseudintermedius* strain 222 cultivation.**

→


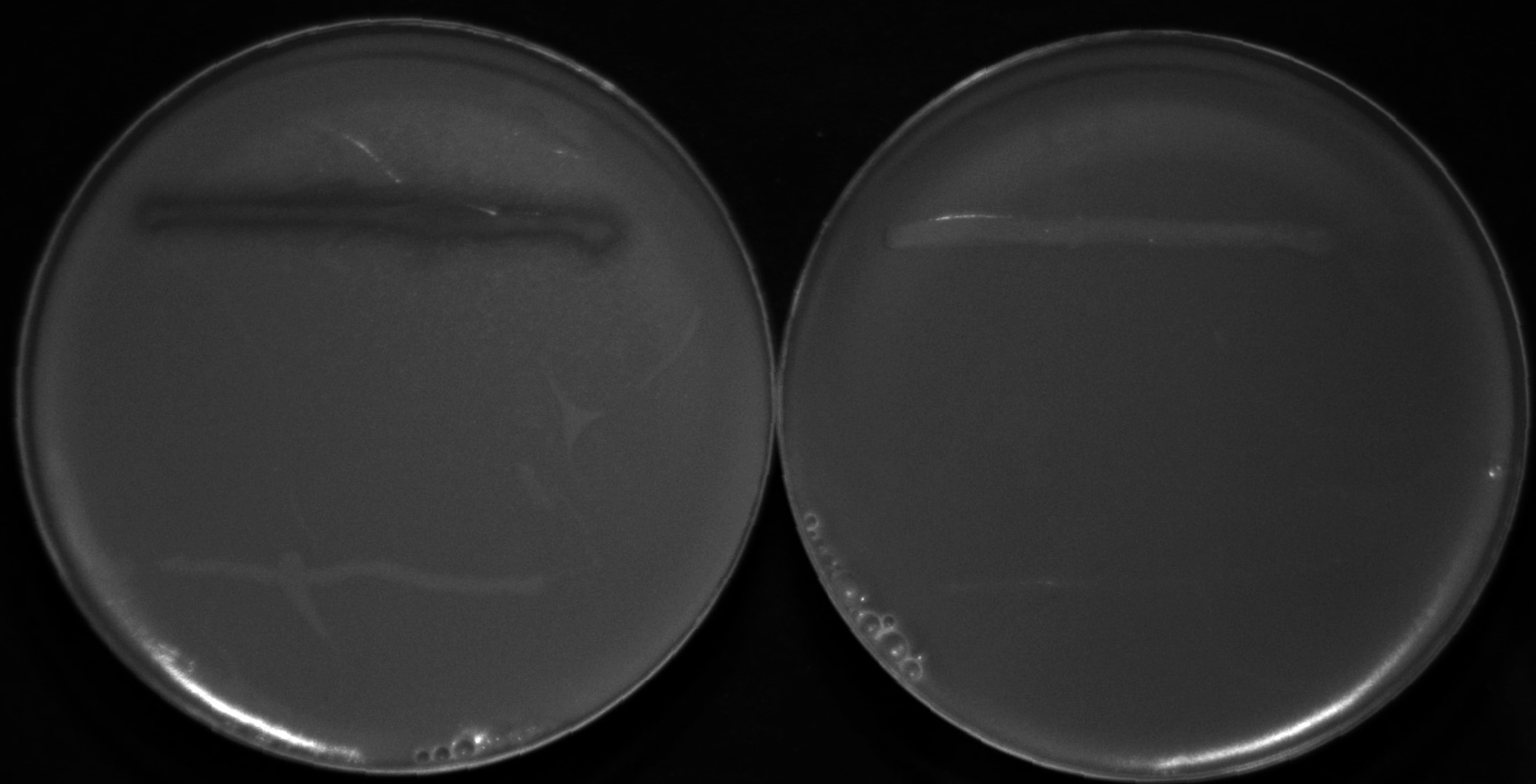


22219 222

222→ 222→

22219→ 22219→

**Supplementary Figure S5.** **Microbiological assay illustrating that the producer strain, *Staphylococcus pseudintermedius* 222, is resistant to the bactericidal action of BacSp222.** On two soft agar nutrient plates containing the suspension of *Staphylococcus pseudintermedius* 222 (a producer of BacSp222) or LMG 22219 (a strain that does not produce bacteriocins, susceptible to BacSp222), two horizontal streaks of both *S. pseudintermedius* 222 and 22219 were made, and the plates were incubated overnight at 37°C. The clear zone around strain 222 grown on the plate containing strain 22219, and the lack of a corresponding zone around strain 222 grown on the plate containing *S. pseudintermedius* 222 confirmed that the producer cells were resistant to the toxic effects of the secreted bacteriocin.


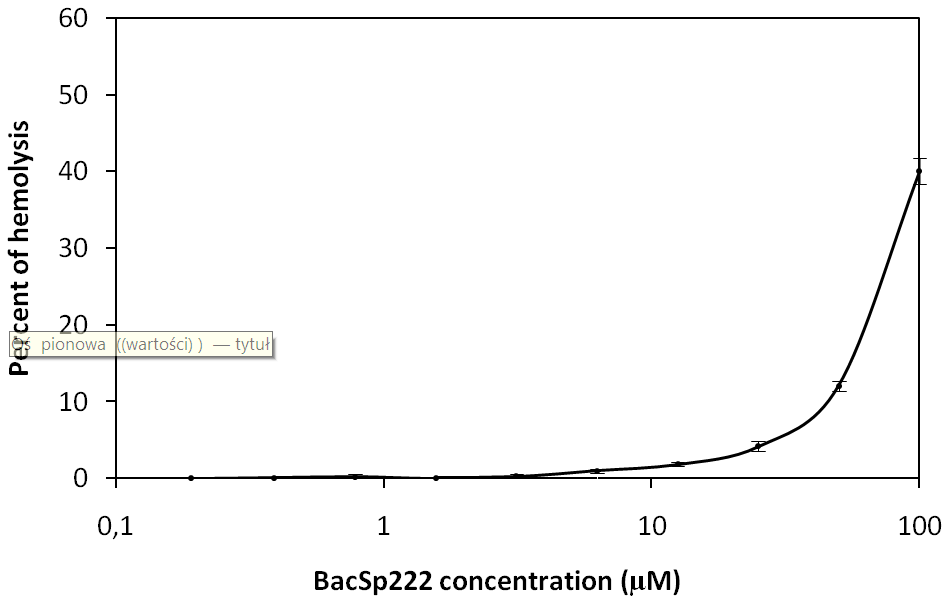


0.1

**Supplementary Figure S6.** **The haemolytic activity of BacSp222 against human erythrocytes.** The degree of haemolysis is expressed as the percent of haemoglobin release relative to complete lysis of erythrocytes by a detergent (1% SDS). The points represent the average values from three independent experiments ± SD.

**LDH**

**MTT**

**Supplementary Figure S7.** **Cytotoxic activity of BacSp222 against HeLa and ASC cells, as evaluated by LDH and MTT assays.** LD50 values are provided in the inserts. The bars represent the average values from three independent experiments ± SD.
